# Transcutaneous Carbon Dioxide Therapy Significantly Accelerates Diabetic Foot Ulcer Healing: A Multicentre Randomised Controlled Trial

**DOI:** 10.3390/biomedicines14020422

**Published:** 2026-02-13

**Authors:** Igor Frangež, Miloš Potkonjak, Skender Veliu, Željko Metelko, Anica Badanjak, Julija Križaj, Helena Ban Frangež, Tamara Poljičanin, Marco Meloni, Nikolaos Papanas, Tomislav Bulum

**Affiliations:** 1 Department of Surgical Infections, University Medical Centre Ljubljana, Zaloška Cesta 2, 1000 Ljubljana, Slovenia; igor.frangez@kclj.si (I.F.); julija.krizaj@hotmail.com (J.K.); 2 Faculty of Medicine, University of Ljubljana, Vrazov Trg 2, 1000 Ljubljana, Slovenia; helena.ban@kclj.si; 3 Department of Vascular Surgery, General Hospital Novo Mesto, Šmihelska Cesta 1, 8000 Novo Mesto, Slovenia; milospotkonjak@gmail.com; 4 Department of Surgery, General Hospital Ptuj, Potrčeva Cesta 23, 2250 Ptuj, Slovenia; skender.veliu@gmail.com; 5 Polyclinic for Physical Medicine and Rehabilitation, Kalinovica 3, 10000 Zagreb, Croatia; metelko.zeljko@gmail.com (Ž.M.); anica.badanjak@gmail.com (A.B.); 6 Department of Obstetrics and Gynaecology, University Medical Centre Ljubljana, Slajmerjeva ul. 3, 1000 Ljubljana, Slovenia; 7 Zagreb County Health Centre, Ljudevita Gaja 37, 10430 Samobor, Croatia; tamara.poljicanin@gmail.com; 8 Department of Systems of Medicine, University of Tor Vergata, 00133 Rome, Italy; meloni.marco@libero.it; 9 Department of Medical Sciences, Division of Endocrinology and Diabetology, University Hospital Fondazione Policlinico Tor Vergata, 00133 Rome, Italy; 10 Second Department of Internal Medicine, Democritus University of Thrace, Diabetes Centre-Diabetic Foot Clinic, 69100 Alexandroupolis, Greece; papanasnikos@yahoo.gr; 11 Vuk Vrhovac University Clinic for Diabetes, Endocrinology and Metabolic Diseases, Clinical Hospital Merkur, Dugi Dol 4a, 10000 Zagreb, Croatia; 12 School of Medicine, University of Zagreb, Šalata 3, 10000 Zagreb, Croatia

**Keywords:** transcutaneous CO_2_ therapy, diabetic foot ulcer, wound healing, chronic wound

## Abstract

**Background/Objectives:** Diabetic foot ulcers (DFUs) represent common and severe complications of diabetes mellitus (DM). The aim of our parallel-group, open-label, superiority, multicentre randomised controlled trial (RCT) was to evaluate the effectiveness of transcutaneous gaseous carbon dioxide therapy (hereinafter CO_2_ therapy) in ulcer healing in patients with non-healing DFUs. **Methods:** A total of 115 participants (89 males and 26 females, aged 65.7 ± 10.9 years) with a non-healing DFU were randomised to the intervention (76 participants) and control (39 participants) group. Participants in the intervention group received standard of care, combined with CO_2_ therapies administered every weekday for four consecutive weeks. Participants in the control group received only standard of care. **Results:** After 4 weeks, 49 (64.5%) ulcers in the intervention group healed, compared with three (7.7%) in the control group (primary outcome). The percentage ulcer area reduction from baseline (secondary outcome) was significantly larger (*p* ≤ 0.001) in patients from the intervention group, with the median value 100% (18.4–100%), compared to the patients from the control group, with the median value 40% (−300–100%). Patients receiving CO_2_ therapy had 35.4-fold higher odds of ulcer healing versus controls (OR 35.4, 95% CI 6.68–187.53; *p* < 0.001), and in multivariate logistic regression, CO_2_ therapy remained independently associated with healing, while larger baseline ulcer area was associated with lower odds of healing (OR 0.654, 95% CI 0.438–0.977; *p* = 0.038). No adverse effects were reported. **Conclusions:** CO_2_ therapy significantly contributes to ulcer healing in patients with non-healing DFUs, with no observable adverse effects, demonstrating significant potential as an effective and safe complementary treatment of DFUs in conjunction with standard of care.

## 1. Introduction

Diabetes mellitus (DM) is one of the fastest-growing global health emergencies nowadays, and it already represents a major public health crisis worldwide. The International Diabetes Federation states in their 2025 release of Diabetes Atlas that the number of people with DM will increase from 588.7 million in 2024 to 852.5 million in 2050, representing a 45% increase [1]. DM is characterised by numerous complications, of which diabetic foot ulcers (DFUs) are one of the major ones. DFUs are associated with reduced quality of life, high levels of morbidity and mortality, and economic burden [2]. Given the lifetime incidence rate of DFUs of 19–34% [3] and the projected number of people with a DFU for 2050 [1], a total of up to 280.7 million people worldwide will be diagnosed with DFUs. Furthermore, considering that the recurrence rate of DFUs is 40% within a year and 60% within 3 years after ulcer healing [3], DFU prevention and management are of high importance to the global society.

Although prevention is always better than treatment, preventing DFUs is challenged by adherence issues, limited availability of trained multidisciplinary teams, and the complex, multifactorial pathophysiological mechanisms underlying DFUs. As a result, numerous interventions aimed at enhancing DFU healing have been proposed. While some have already been recognised by the International Working Group on the Diabetic Foot (IWGDF) [4], many promising interventions are being studied worldwide. One such intervention is the transcutaneous application of gaseous carbon dioxide (CO_2_) (hereinafter CO_2_ therapy). In this intervention technique, therapeutic concentrations of medical-grade CO_2_ are applied to the skin’s surface in a safe, non-invasive manner. The main therapeutic mechanism is derived from the human body’s natural response to locally increased CO_2_ concentration. The key responses are the Bohr effect [5] and the vasodilatory effect of CO_2_ [6,7]. CO_2_ therapy activates the Bohr effect by significantly decreasing intracellular pH via absorbed CO_2_, which reduces haemoglobin’s affinity for oxygen and facilitates its dissociation in local tissues [5]. Because vasodilatory capacity is impaired in diabetic foot, these mechanisms enhance local microvascular perfusion, thereby improving tissue oxygenation in patients with DFUs. Furthermore, although the researchers primarily focus on CO_2_ therapy as an intervention method, its efficacy is also suggested in preventing DFUs [8]. The promising nature of CO_2_ therapies is also evidenced by recent reviews [9,10,11].

The objective of our multicentre randomised controlled trial (RCT) was to evaluate whether CO_2_ therapy, in combination with standard of care, is superior to standard of care alone in promoting ulcer healing among patients with DFUs. To our knowledge, this is the first multicentre RCT to investigate this intervention.

## 2. Materials and Methods

The key terminology used throughout this article is aligned with the terminology defined by the IWGDF in their document on definitions and criteria for diabetic foot disease [12] (e.g., DFU, healed foot ulcer, first-ever foot ulcer, recurrent foot ulcer) and the paper by Jeffcoate et al. [13] (e.g., ulcer healing, ulcer healing rate).

Our trial was designed as a parallel-group, superiority, multicentre RCT with an allocation ratio of 2:1. Participants or the public were not involved in the design, conduct, or reporting of this trial. No important/relevant changes were made to the trial’s design, outcomes, or analyses after the commencement of the study. The eligibility/inclusion criteria for our multicentre RCT were as follows:

- Age between 18 and 90 years;
- Confirmed diagnosis of DM Type 1 or Type 2;
- Confirmed diagnosis of a non-healing DFU without clinical signs of infection;
- Being able to provide informed consent for participation;
- Absence of any disease/comorbidity representing a contraindication (deep vein thrombosis, chronic kidney diseases—Grade IV, chronic heart failure—NYHA Class IV, malignant diseases, systemic infection with elevated inflammatory markers, osteomyelitis) for the CO_2_ therapy.

Beyond failing to meet the inclusion criteria presented above, participants were excluded based on the following additional exclusion criteria:

- Severe ischemia/chronic limb-threatening ischemia requiring urgent vascular specialist assessment or planned revascularisation;
- Clinically infected ulcer, with infection severity assessed using the IWGDF classification;
- Suspected PAD requiring diagnostic vascular assessment (e.g., non-palpable dorsalis pedis and posterior tibial pulses) in the absence of prior vascular documentation.

A non-healing DFU was defined as an ulcer with a duration of at least 1 month without a 50% decrease in ulcer area despite management in a specialist centre following standard of care [14]. Subjects who met all eligibility criteria were then invited to the trial by medical professionals. The four participating institutions in the trial were the Department of Surgical Infections, University Medical Centre Ljubljana (Ljubljana, Slovenia), the Department of Vascular Surgery, General hospital Novo mesto (Novo mesto, Slovenia), the Department of Surgery, General hospital Ptuj (Ptuj, Slovenia), and the Polyclinic for Physical Medicine and Rehabilitation with Physical Therapy and Vascular Surgery, Neurology, Endocrinology and Diabetology (Zagreb, Croatia). The trial, including recruitment and follow-up, was conducted between June 2023 and December 2024.

During the invitation process, subjects satisfying the eligibility criteria were presented with the trial procedure, their rights, including the right to withdraw from the trial at any time for any reason, and their obligations, such as adherence to the trial’s schedule and protocol. After the initial invitation, the subjects were given the opportunity to ask questions about any aspect of the trial. Finally, the subject’s willingness to be enrolled in our RCT was confirmed by signing the informed consent form.

Participants willing to take part in the trial were first assessed for eligibility and then randomised in a 2:1 ratio to the intervention or control group. The random allocation sequence was generated in a custom Microsoft Excel (Microsoft, Redmond, United States) worksheet using the permuted block randomisation method with a fixed block size of 3 by an independent person not involved in recruitment or outcome assessment. Allocation concealment was ensured using sealed, opaque, sequentially numbered envelopes, preventing personnel enrolling participants from foreseeing the next assignment. Regardless of group allocation or participating institution, identical standard wound care was performed consistently, comprising debridement, local ulcer care, selection of appropriate dressings, infection management protocols, and prescription of off-loading shoes (if not already used by a participant), aligned with the IWGDF Guideline on interventions to enhance healing of foot ulcers in persons with DM [4]. Ulcer dressing changes were performed three times weekly for all participants, regardless of group allocation. Participants in the intervention group underwent 50 min CO_2_ therapy once per working day for 4 consecutive weeks, for a total of 20 therapies. The only difference between the intervention and control groups was, therefore, the administration of CO_2_ therapy. The rationale for administering CO_2_ therapy every weekday for 4 weeks was based on the established clinical protocol and pathway for DFUs at the University Medical Centre Ljubljana. To support treatment adherence and minimise loss to follow-up, reminder calls/SMS messages and transport to and from the clinic were provided to participants in both groups as needed. At baseline, the following data were collected for each participant: gender, age, DM type, previously diagnosed peripheral arterial disease (PAD) and distal sensory polyneuropathy (DSPN), ulcer type, ulcer classification, ulcer duration, and ulcer area. The presence of PAD and DSPN was determined based on documented medical history. The diagnosis of PAD needed to be made by an angiologist via duplex ultrasound, and the diagnosis of DSPN by a neurologist (lack of protective sensation during a 12-point 10 g monofilament test and diminished perception of vibration on the big toe using a 128 Hz tuning fork). Accordingly, ulcers were classified as neuropathic (DSPN only), ischemic (PAD only), or neuroischemic (concomitant DSPN and PAD). The ulcer classification was performed using the Falanga wound bed classification (ABCD), which stratifies ulcers based on the quality of the wound bed and periwound tissue (Score A: The wound is 100% covered in healthy granulation tissue with no fibrin or exudate present. Score B: Granulation is high (50–100%), fibrin has begun to form, and there is no exudate. Score C: Granulation is below 50%, fibrin is present, and is no exudate. Score D: Regardless of the amount of granulation, both fibrin and exudate are present.) [15]. The ulcer area was defined using a non-contact 3D laser scanner (DAVID SLS-2, David Vision Systems GmbH, Koblenz, Germany).

Formal ulcer assessment was performed at each participating institution at baseline and after 4 weeks. Ulcer healing after 4 weeks was defined as full epithelialisation of the ulcer surface, without drainage (i.e., no exudate) and without the need for dressings, verified across two sequential follow-up visits every 2 weeks. The unhealed ulcers were reassessed using ulcer classification and ulcer area after 4 weeks.

The CO_2_ therapies were administered by trained nurses using a PVR system^®^ medical device (Derma Art d.o.o. and PVR med d.o.o., Brežice, Slovenia). They were carried out as follows: the participant’s lower body was first isolated in a single-use, biocompatible polyethene/polyethylene therapeutic wrap, sealed at the waist. Second, the PVR system inflated the wrap with medical-grade CO_2_ until a 99.9% concentration of CO_2_ was achieved. Once done, the CO_2_ therapy session began and lasted for 50 min. During the therapy, the subjects were in a supine position. After the completion of CO_2_ therapy, CO_2_ was pumped out of the wrap, and the wrap was removed from the participants. During and after each therapy session, participants were instructed to report any symptoms they experienced, which could help the researchers identify potential adverse effects of CO_2_ therapy.

All potential adverse effects/harms that could appear during a CO_2_ therapy session were assessed by medical personnel observing participants for local adverse reactions, such as skin irritation or oedema. These observations were facilitated by transparent therapeutic wraps, which enabled continuous visual assessment of the skin throughout the session. Additionally, personnel remained vigilant in observing participant behaviour for any non-verbal signs of discomfort or physiological distress during the procedure. To evaluate systemic effects, participants were instructed to immediately report any subjective symptoms, including headache, dizziness, tinnitus, chest pain, palpitations, or fatigue. Finally, at each participant visit, a physical examination of the ulcer site, together with a non-systematic assessment (performed through patient reporting) of any adverse events between scheduled visits, was conducted. Vital signs were not monitored before or after the CO_2_ therapy sessions, as previous studies investigating the systemic effects of CO_2_ therapies found no clinically relevant changes in heart rate or arterial blood pressure [6,7].

The primary outcome was the absolute number of healed ulcers in 4 weeks (assessed by visual inspection of a trained professional verified across two sequential follow-up visits every two weeks). The secondary outcome was the percentage ulcer area reduction after 4 weeks compared with baseline (evaluated by measuring ulcer area at baseline and after 4 weeks). The normality of the distributions was tested using the Shapiro–Wilk W test, and the homogeneity of variances was tested using the Levene test. Differences between groups in continuous variables were analysed using the Mann–Whitney U test. Differences in the prevalence of individual conditions were compared using the chi-square test and Yates’ corrected chi-square, where appropriate. Multivariate (binary) logistic regression was performed to predict the probability of ulcer healing after 4 weeks. Statistical significance was defined as *p* < 0.05. All statistical analyses were carried out using IBM SPSS Statistics V21.0 (IBM, New York, NY, USA). The trial yielded a complete dataset as all enrolled participants successfully completed the trial’s protocol. Only individuals with required baseline data were eligible for inclusion; any candidates with missing initial data were excluded prior to enrolment. Consequently, no data imputation methods were required.

This study was conducted and reported in accordance with the CONSORT 2025 guidelines. The trial is registered at ClinicalTrials.gov (NCT04561609; https://clinicaltrials.gov/study/NCT04561609), with a registration date of 6 September 2020. The trial protocol, statistical analysis plan, individual de-identified participant data, statistical code, and other relevant materials are available from the corresponding author upon reasonable request.

## 3. Results

**Figure 1:**
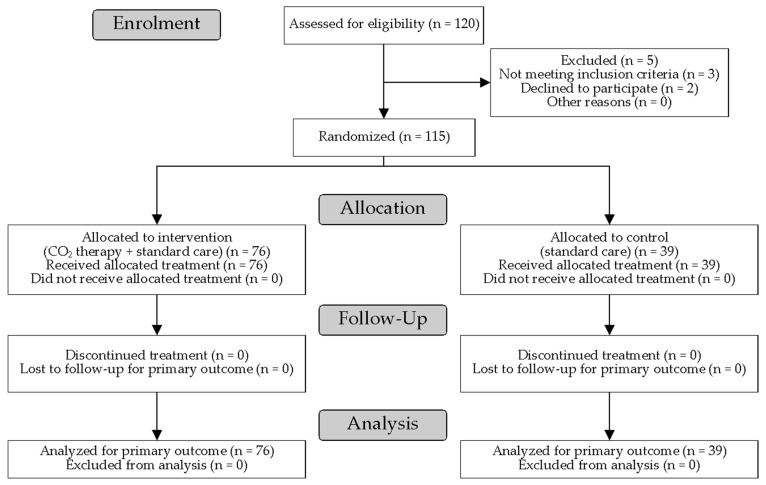
The flow diagram of participant progress through the trial.

Figure 1 shows the flow diagram of participant progress through our RCT.

A total of 120 Caucasian participants were enrolled in our study, of whom five were excluded for not meeting the eligibility criteria, leaving 115 participants (89 males and 26 females, aged 65.7 ± 10.9 years) randomised to the intervention and control groups. All randomised participants received the allocated treatment and completed the 4-week assessment; there were no losses to follow-up and no exclusions from the intention-to-treat analysis. The participants were randomly allocated to the intervention and control groups at a 2:1 allocation ratio. The characteristics of the two groups are presented in Table 1. There were no statistically significant differences in participants’ gender, age, and BMI between the intervention and control groups.

Table 2 shows the ulcer characteristics of participants from both groups. There were no statistically significant differences in ulcer type, ulcer classification, or baseline ulcer area, whereas ulcer duration was significantly shorter in the intervention group than in the control group. All ulcers were located on the plantar side of the forefoot or the plantar side of the midfoot. After 4 weeks, ulcer healing (primary outcome) was achieved in 64.5% of all ulcers in the intervention group (49 out of 76) and 7.7% in the control group (3 out of 39). Consequently, the ulcer area after 4 weeks was significantly smaller in the intervention group than in the control group, and the percentage ulcer area reduction was significantly greater in the intervention group than in the control group (secondary outcome).

The Falanga scores of unhealed ulcers differed significantly between the intervention and control groups, with most ulcers in the intervention group having a score of A and those in the control group having a score of B.

Table 3 presents the results of the comparison of percentage ulcer area reduction between the two groups, expressed as a function of the baseline Falanga score. In the intervention group, median values are comparable across different Falanga scores, whereas there are observable differences in median percentage reductions in ulcer area from the control group.

In both groups, no systemic adverse effects, ulcer infections, amputations, or other surgical interventions were observed. Participants in the intervention group reported no adverse sensations or treatment-related discomfort throughout the trial. Moreover, descriptive, participant-reported outcomes were uniformly positive, with reports of reduced pain and decreased unpleasant paraesthesia, such as tingling, numbness, and burning sensations; no objective verification of pain reduction or paraesthesia reduction was performed. In the control group, worsening of an ulcer appeared in four ulcers out of 39 (10.2%). The intervention (CO_2_ therapy plus standard of care) and comparator (standard of care) were administered exactly as described in the trial protocol. None of the participants enrolled in the trial discontinued participation prematurely.

The multivariate logistic regression model for the outcome of the ulcer healing after 4 weeks (*p* < 0.001, Nagelkerke pseudo R-square = 55.1%) revealed that an increase in baseline ulcer area was associated with a decrease in the odds of the ulcer healing after 4 weeks, with an odds ratio of 0.654 and CO_2_ therapy being associated with an increase in odds of ulcer healing after 4 weeks, with an odds ratio of 35.392 (Table 4). Other included variables were not significant predictors of ulcer healing after 4 weeks.

## 4. Discussion

Achieving ulcer healing in the intervention group (49 out of 76 ulcers or 64.5% of all ulcers) is the key result of our trial. The ulcer healing achieved in our study is comparable to that reported in previous studies on the utilisation of CO_2_ therapies for ulcer healing by Macura et al. (66.7% of 30 ulcers) [16] and Bulum et al. (67.5% of 40 ulcers) [17]. A direct comparison of the results is fully justified, provided they used the same intervention protocol (twenty 50 min-long CO_2_ therapy sessions per workday for a total of 4 weeks) as we did in our trial. The extra dimension of our trial, which further contributes to the research on CO_2_ therapies, is that it was conducted at multiple centres across two countries. This is important for several reasons. First, multiple trained medical professionals administered CO_2_ therapy, but there was no overall effect on ulcer healing, indicating that CO_2_ therapy is highly repeatable. Second, there were likely psychosocial factors affecting participants’ adherence to the standard of care, which might differ across countries and socio-economic areas [18,19]. Third, although the standard of care was uniform across all participating institutions, potential minor variability in its execution due to organisational differences might have occurred. Despite the variations presented, the results are comparable to those reported in single-centre studies. The therapeutic potential of CO_2_ therapy in ulcer healing, commonly mediated by improved perfusion, was recently synthesised in a scoping review by Prazeres et al. [11]. In addition, a recent study by Yang et al. [9] comparing gas-based therapies for DFUs reported that CO_2_ therapy achieved the highest surface under the cumulative ranking curve (SUCRA) rankings for both healing rate (SUCRA of 0.998) and ulcer area reduction (SUCRA of 0.828). However, these promising estimates relied on a single CO_2_ therapy-based trial. Notably, Bulum et al. [17] recently reported favourable ulcer healing outcomes and improvements in protective sensation following transcutaneous CO_2_ therapy; this study (not covered in the reviews presented above) further strengthens the clinical evidence base. Our multicentre RCT contributes new evidence that corroborates the therapeutic rationale and extends prior findings, indicating that CO_2_ therapy offers healing rates that potentially exceed those typically reported for standard of care and commonly used adjunctive modalities.

Even more important than the number of healed ulcers in the intervention group is the rapidity of healing within 4 weeks, which represents a marked improvement compared with healing outcomes typically reported in the literature. For example, Coye et al. [20] report in their meta-analysis of randomised controlled trials on ulcer healing in patients with DFUs by means of standard-of-care interventions that, within a 12- to 24-week treatment period, the healing rate was 33.15%, and the average healing time was 50.14 ± 31.10 days. Armstrong et al. [21] report a healing rate of 30% to 40% after 12 weeks. The significant improvement in ulcer healing in our trial is further supported by the absence of observable side effects, ulcer infections, or amputations among participants in the intervention group. Furthermore, participants reported reduced paraesthesia (including numbness, tingling, and stabbing sensations) and diminished pain, highlighting the benefits of CO_2_ therapy and its potential to improve patients’ quality of life. Moreover, ulcer healing was observed in participants whose ulcers were classified as ischemic or neuroischemic. This observation suggests that CO_2_ therapy may enhance microvascular perfusion, as reported by Finžgar et al. [6,7], including in patients with a previously documented PAD diagnosis, potentially mitigating the functional perfusion deficit at the ulcer level; however, our trial lacks data to support this, except for the Falanga scores. A significant transition of Falanga toward healthy granulation in the intervention group indicates a qualitative improvement in the wound bed that facilitates rapid epithelialisation within the 4-week timeframe.

Regarding the ulcers in the intervention group that remained unhealed after 4 weeks, it is crucial to note that the area of 20 of 27 of these ulcers decreased by over 50%. This significant reduction indicates that they should no longer be categorised as non-healing ulcers, which was their baseline status. Conversely, the area of five ulcers in the control group increased over the same 4-week period. The observed deterioration underscores the refractory nature of these ulcers under the standard of care in place.

Another important strength of our trial is the use of a multivariate logistic regression model to analyse outcomes at 4 weeks, including ulcer healing and improvement in the wound bed (ulcer classification). The fact that CO_2_ therapy was the most significant predictor of an increase in the odds of ulcer healing after 4 weeks (*p* < 0.001) provides a strong foundation for further studies, which should incorporate as many relevant details about the person and ulcer characteristics that affect ulcer healing in order to improve the reliability of the multivariate logistic regression model.

The 2:1 allocation ratio was selected to maximise experience with CO_2_ therapy, particularly across the two centres implementing it for the first time, to accumulate sufficient practical experience and to provide a good basis for estimating the incidence of potential adverse effects. In summary, this allocation rate enabled all centres to thoroughly characterise the risk profile of CO_2_ therapy.

Despite the clear advantages of our trial presented above, there are some limitations we are aware of. First, when it comes to the statistically significant difference in ulcer duration between the groups (median 5 months vs. 8 months), we still followed the core principle of RCTs, i.e., the intention-to-treat principle, treating this difference as a result of chance. Crucially, the multivariate logistic regression analysis did not confirm that ulcer duration was a significant predictor of healing, ruling it out as a confounding factor. Second, due to the visibly distinct nature of the interventions, blinding of participants and care providers was not feasible, resulting in an open-label randomised controlled design. Third, there was a disparity in visit frequency between the two groups. While the participants from the intervention group attended daily sessions for CO_2_ therapy administration, the participants from the control group visited their corresponding institution only three times per week for standard of care. Although this could theoretically introduce attention bias, we strictly mitigated this by prohibiting any ulcer manipulation, debridement, or dressing changes during the CO_2_ therapy-only visits. Therefore, the improved healing outcomes observed in the intervention group are attributable to the physiological effects of the CO_2_ therapy rather than increased ulcer surveillance or hygiene. Nevertheless, the differing visit frequencies, together with the aforementioned open-label design, may have introduced performance or attention bias. Consequently, both factors must be considered when interpreting the magnitude of the observed effects. Fourth, an a priori sample size or power calculation was not performed, potentially precluding the detection of subtle yet clinically significant differences. Consequently, non-significant findings should be interpreted with caution rather than as a definitive absence of effect. While a 2:1 allocation ratio may introduce greater uncertainty and potentially reduce regression stability, it is not expected to bias effect size estimates. Nonetheless, a cautious interpretation of our findings is essential. Next, the reported reductions in pain and improvements in paraesthesia were not validated using standardised assessment tools; therefore, these self-reported findings should be interpreted with caution. Next, as a pragmatic clinical trial, the sample size was determined by feasibility considerations rather than a formal a priori power calculation. Recruitment targets were based on the expected number of eligible patients across the four participating centres, within the constraints of clinical and organisational resources. Finally, our study population was limited to Caucasian participants. Although the foundational physiological mechanisms underlying transcutaneous CO_2_ administration are theoretically universal, the generalisability of these findings across diverse ethnic groups remains to be established.

Our future work is aimed toward (1) carrying out a blinded multicentre RCT by including participants of various ethnicities, (2) providing further information on recurrence rate and other long-term outcomes in DFUs healed with CO_2_ therapy (Bulum et al. reported recurrence rate of 17.5% after 1 year [17]), and (3) evaluating cost–benefit/cost-effectiveness of CO_2_ therapies. The first aim is important because there is a lack of research on CO_2_ therapies for DFU management (or prevention) involving other ethnicities. This topic will become increasingly relevant, as the highest expected increases in DM patients are projected for the period from 2024 to 2050 in Africa (142% increase), followed by the Middle East and North Africa (92% increase) and Southeast Asia (73% increase) [1]. Furthermore, large-scale confirmatory RCTs would be an important next step to validate these findings and provide robust evidence to support broader clinical adoption. Our second future work is an extensive study of the recurrence rate of healed ulcers treated with CO_2_ therapy. CO_2_ therapies are reported to have a positive effect on impaired microcirculatory function [7] and consequent neuropathy [8] in patients with DM. Since DM is associated with microvascular disease that leads to the formation of DFUs, the potential of CO_2_ therapies to reduce recurrence rates would represent another major advantage. Finally, as with any other treatment method, cost–benefit/cost-effectiveness analyses of CO_2_ therapies for DFU healing represent another crucial step in implementing CO_2_ therapies in healthcare systems.

## 5. Conclusions

The key result of our multicentre RCT is a 64.5% healing rate within 4 weeks, which represents a highly promising outcome supporting the use of CO_2_ therapy as a complementary intervention to the standard of care for patients with DFUs. Specifically, CO_2_ therapy appears to be a very useful adjuvant for managing non-infected neuroischemic DFUs that do not improve despite the best standard of care; however, these findings should be viewed in light of our trial’s short follow-up period and open-label design. This efficacy is further strengthened by the absence of systemic side effects, ulcer infections, amputations, or additional surgical interventions in the intervention group. Finally, descriptive, participant-reported outcomes were uniformly positive (reduced pain and decreased unpleasant paraesthesia).

## Figures and Tables

**Table 1:** Person characteristics of the control and intervention groups.

Variable	Control Group	Intervention Group	*p*
Group size	39	76	/
Age, mean ± SD [year]	63.97 ± 11.45	66.63 ± 10.52	0.558
Gender	M: 30 (76.9%) F: 9 (23.1%)	M: 59 (77.6%) F: 17 (22.4%)	0.931
BMI, mean ± SD [kg/m^2^]	30.27 ± 5.14	30.05 ± 4.57	0.818
DM type	Type 1: 0 (0.0%) Type 2: 39 (100.0%)	Type 1: 2 (2.6%) Type 2: 74 (97.4%)	/
Previously diagnosed PAD	Yes: 21 (53.8%) No: 18 (46.2%)	Yes: 49 (64.5%) No: 27 (35.5%)	0.269

Legend: Normally distributed variables are expressed as mean ± SD. SD: standard deviation, *p*: *p*-value, M: male, F: female, BMI: body mass index, DM: diabetes mellitus, PAD: peripheral arterial disease.

**Table 2:** Ulcer characteristics of the control and intervention groups.

Variable	Control Group	Intervention Group	*p*
No. of ulcers	39	76	/
Duration of ulcer, median (min–max) [month]	8 (1–50)	5 (1–144)	0.019
Ulcer type	I: 5 (12.8%) N: 18 (46.2%) NI: 16 (41.0%)	I: 24 (31.6%) N: 27 (35.5%) NI: 25 (32.9%)	0.09
Ulcer area at baseline, median (min–max) [cm^2^]	1.50 (0.20–14.10)	1.47 (0.30–11.09)	0.719
Ulcer area after 4 weeks, median (min–max) [cm^2^]	0.80 (0.00–9.13)	0.00 (0.00–2.89)	<0.001
Ulcer classification at baseline (Falanga score)	A: 3 (7.7%) B: 19 (48.7%) C: 12 (30.8%) D: 5 (12.8%)	A: 1 (1.3%) B: 50 (65.8%) C: 24 (31.6%) D: 1 (1.3%)	0.072 *
No. of healed ulcers	3 (7.7%)	49 (64.5%)	/
Ulcer classification after 4 weeks (Falanga score) *	A: 4 (10.3%) B: 21 (53.8%) C: 11 (28.2%) D: 0 (0.0%)	A: 23 (30.3%) B: 4 (5.2%) C: 0 (0.0%) D: 0 (0.0%)	<0.001 *
Percentage ulcer area reduction, median (min–max) [%]	40.00 (−300.00–100.00)	100.00 (18.37–100.00)	<0.001

Legend: Non-normally distributed ones as median (min–max). *p*: *p*-value, N: neuropathic, NI: neuroischemic, I: ischemic, * Yates’s chi-square.

**Table 3:** Percentage ulcer area reduction as a function of a baseline Falanga score.

	Percentage Ulcer Area Reduction, Median (Min–Max) [%]		
Falanga Score at Baseline	Control Group	Intervention Group	*p*
A	18.00 (10.00–100.00)	91.78 (91.78–91.78)	N/A
B	38.00 (−300.00–100.00)	100.00 (18.37–100.00)	<0.001
C	49.50 (−10.00–100.00)	97.97 (43.75–100.00)	<0.001
D	72.00 (−100.00–77.00)	97.39 (97.39–97.39)	N/A

Legend: Non-normally distributed variables are presented as median (min–max). *p*: *p*-value, N/A: not available due to the small number of ulcers with Falanga scores A and D.

**Table 4:** Multinominal logistic regression model for the outcome of ulcer healing after 4 weeks.

Parameter	OR	95% CI	*p*
Patient age	1.044	0.987–1.105	0.129
Gender	1.478	0.437–4.998	0.530
BMI	1.116	0.978–1.273	0.104
Duration of ulcer	0.991	0.962–1.019	0.516
Ulcer area at baseline	0.654	0.438–0.977	0.038
Ulcer type NI	0		
Ulcer type I	3.355	0.827–13.620	0.090
Ulcer type N	1.182	0.328–4.252	0.798
CO_2_ therapy	35.392	6.679–187.529	<0.001
Falanga B vs. C	2.794	0.825–9.466	0.099

Legend: BMI—body mass index, CI—confidence interval, OR—odds ratio, *p*—*p*-value. Only ulcers with Falanga scores B and C were included in the model due to the small number of ulcers with Falanga scores A and D.

## Data Availability

The original contributions presented in this study are included in the article. Further inquiries can be directed to the corresponding author.

## Abbreviations

The following abbreviations are used in this manuscript:

BMI	body mass index
CI	confidence interval
CO_2_	carbon dioxide
DFU	diabetic foot ulcer
DM	diabetes mellitus
DSPN	distal sensory polyneuropathy (DSPN)
F	female
I	ischemic
IWGDF	International Working Group on the Diabetic Foot
M	male
N	neuropathic
NI	neuroischemic
p	p-value
PAD	peripheral arterial disease
RCT	randomised controlled trial
SUCRA	surface under the cumulative ranking curve
